# Experimental Models of Irritable Bowel Syndrome and the Role of the Enteric Neurotransmission

**DOI:** 10.3390/jcm7010004

**Published:** 2018-01-03

**Authors:** Maria Giuliana Vannucchi, Stefano Evangelista

**Affiliations:** Department of Experimental and Clinical Medicine, Research Unit of Histology and Embryology, University of Florence, Viale Pieraccini, 6-50139 Florence, Italy; stefano.evangelista55@virgilio.it

**Keywords:** corticotropin releasing factor (CRF), irritable bowel syndrome (IBS), maternal separation (MS), neurotransmitters, pain, psychosocial stress, visceral hyperalgesia, water avoidance stress (WAS), wrap restrain stress (WRS)

## Abstract

Irritable bowel syndrome (IBS) is one of the most common gastrointestinal diseases in humans. It is characterized by visceral pain and/or discomfort, hypersensitivity and abnormal motor responses along with change in gut habits. Although the etio-pathogenesis of IBS is only partially understood, a main role has been attributed to psychosocial stress of different origin. Animal models such as neonatal maternal separation, water avoidance stress and wrap restraint stress have been developed as psychosocial stressors in the attempt to reproduce the IBS symptomatology and identify the cellular mechanisms responsible for the disease. The study of these models has led to the production of drugs potentially useful for IBS treatment. This review intends to give an overview on the results obtained with the animal models; to emphasize the role of the enteric nervous system in IBS appearance and evolution and as a possible target of drug therapies.

## 1. Introduction

Irritable bowel syndrome (IBS) is a highly prevalent (12–20% in western countries) and multifactorial gastrointestinal disorder [1] whose symptoms are mainly referred to the large bowel. Clinically the IBS is considered a chronic disease characterized by the alternation of remission periods and periods of symptoms exacerbation [2]. Several pathophysiological mechanisms may contribute to IBS manifestations; current concepts attribute an important role to visceral hypersensitivity. In colonic mucosal biopsies of IBS patients it has been shown an increase in activated lymphocytes, mast cells, neutrophilic [3,4] and eosinophilic granulocytes [5] suggesting that inflammation might play a role in IBS hyperalgesia. However, in randomized, double-blind, placebo-controlled trials, the administration of anti-inflammatory drugs, while decreasing the infiltration of immune cells, including mast cells, in the mucosal biopsies, had no significant effect on abdominal pain, bloating or bowel habits, thus leading to reconsider the role of inflammation in IBS hyperalgesia genesis [6,7]. Indeed, although IBS is commonly listed as a functional disorder, the presence of mucosal barrier alterations, low-grade inflammation, brain-gut axis dysfunction and dysbiosis represent evidences for an organic origin of the disease [8].

The etiology of IBS is still poorly known; however, psychosocial stress is the most widely acknowledged risk factor for the development and/or relapse of IBS [9,10]. Notably, up to 80% IBS patients experience comorbid behavioral disorders, such as anxiety or depression [11].

In summary, it is possible that hyperalgesia and psychosocial stressors are able to aliment each other in a vicious circuit responsible for the chronic progression of the disease. The possibility to interrupt this circuit is a challenge for researchers and clinicians.

Psychosocial stress belongs to the exteroceptive stressors [12,13] and are limbic-sensitive and dependent upon the forebrain cognitive circuits mediating the endocrine and autonomic responses to these types of stressors. In the attempt to improve the knowledge of the mechanisms underlining IBS, animal models reproducing psychosocial stressing conditions able to induce some of the IBS main signs, notably hyperalgesia and pain [14,15,16], have been developed. The choice of one or another of these tests is not random since each of them present diverse advantages and limits that have to be weighed before initiating the experimental procedure. 

**Figure 1:**
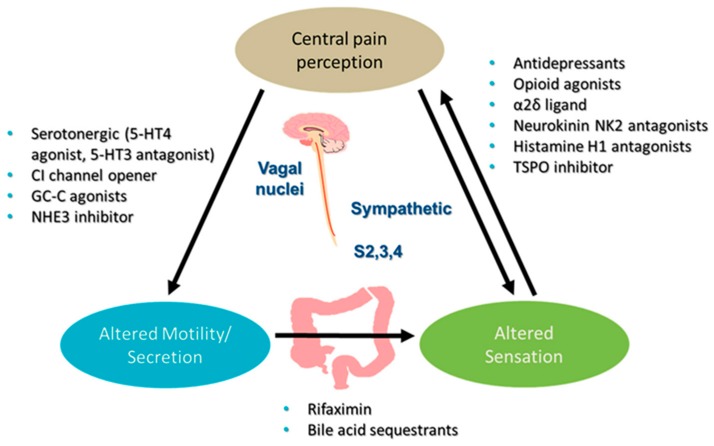
Irritable bowel syndrome (IBS) pharmacotherapy. 5-HT, 5-hydroxy tryptamine; GC-C, guanylate cyclase C; NHE, sodium-hydrogen exchanger; S2, 3 and 4, sacral nerves 2, 3 and 4. Modified from Camilleri and Ford [17].

Presently we will review three of the most used psychosocial stress models: the wrap restrain stress (WRS), an acute stress and two models of chronic stress: the maternal separation (MS) and the water avoidance stress (WAS). In parallel we will focus our attention on those data, obtained by us and other researchers, that highlight the role played by the enteric nervous system in the genesis of IBS symptomatology and the available experimental or clinical pharmacological therapies affecting, directly or indirectly, the enteric neurotransmission (Figure 1).

## 2. Animal Models of IBS

### 2.1. The Wrap Restrain Stress (WRS)

The WRS was introduced in the scientific literature more than 30 years ago as the model for human IBS [18]. Since then, further investigations have partially revised this hypothesis and currently no animal model is considered able to wholly mimic the human disease. The WRS model is commonly applied once (acute test) and consists of a forced immobilization of the animal lasting at least for 2 h. The efficacy of this test is confirmed by the development of an immediate hyperalgesia, quantifiable in colon-rectal distention (CRD) number [19], the inhibition of small intestinal transit, the stimulation of large intestinal activity and increased fecal excretion [18]. We recently demonstrated that rats underwent to the WRS presented a low-grade mucosal inflammation with a significant increase in mast cells and eosinophylic granulocytes [20,21] that overlapped what is described in colonic biopsies of IBS [22]. Moreover, these animals showed important changes in the glial cells, in inhibitory and excitatory neurotransmitters and receptors that were interpreted as responsible for the dysmotility and hypersensitivity present in IBS patients. Therefore, it is reasonable to conclude that WRS represents a suitable model for reproducing, at least in part, the main symptoms present in IBS (i.e., hypersensitivity and dysmotility), and to affirm that WRS shares the face-validity, one of the criteria proposed by Meyer and Collins [23], to validate an animal model. It is a matter of debate whether WRS is adequate to investigate potential drugs [16]. However, WRS misses the most characterizing clinical condition of IBS such as the chronic course. In other words, it does not show good construct-validity [23]. In agreement, when WRS is applied repetitively it triggers adaptive responses [16,24] in opposite to what happens in patients affected by IBS.

### 2.2. Chronic Stressors

Beyond the chronic course, accurate anamneses of IBS patients have attested a significant association between IBS and childhood traumas of different origin or the presence of repeated stress situations in adulthood [25]. Thus, the pre-existence/recurrence of adverse conditions characterize and correlate with the development and maintenance of IBS [8]. These clinical data have moved the researchers to look for animal models carrying chronic rather than acute stressors. Two main models of chronic stress have been proposed to mimic childhood trauma or repeated stress conditions in the adulthood, the maternal separation (MS) and the water avoidance stress (WAS), respectively. These two behavioral tests are distinct not only for the period of application but also for the intensity being the latter considered a mild chronic stress compared to MS. Nevertheless, they share several features, are able to induce a symptomatology similar to that of IBS and reveal sex-differences in sensitivity.

### 2.3. The Maternal Separation (MS)

The stress consists in removing the puppies from the mother for 3 h per day during the first two weeks of life. Since maternal care affects the hypothalamic-pituitary-adrenal (HPA) axis and the cognitive and emotional functions [26], the MS causes stable changes in the central nervous system of these animals [27] At the level of the large intestine, MS promotes, in the adult animals, the development of a condition characterized by visceral hypersensitivity to colorectal distension (CRD) [28,29,30] and colonic mast cell hyperplasia often concentrates close to nerve endings [22], that are two typical signs of IBS [4,31]. Moreover, these animals manifest a hypersensitivity to acute psychosocial stress application, i.e., the WAS applied for 1 h [32].

Interestingly, the experimental data demonstrated a greater sensitivity to MS of females compared to males [16]. Notably, IBS incidence is twofold greater in women than in men and this is true independently of the triggering factors [8]. On the other hand, the potency of this stressor is such that the insurgence of depressive symptoms rather than anxiety is likely [33]. In summary, the MS model possesses either face- or construct- (consistency with the hypothetical pathogenesis) validity [23].

### 2.4. The Water Avoidance Stress (WAS)

Several reports agree that daily chronic stress predicts the severity of the IBS symptoms [34,35,36]. In the light of these observations, rodent models characterized by alternating exposure to stress have been settled and WAS was considered one of the most effective psychological stressors carrying this property. Literature data showed that, in male Wistar rats, a strain with high responsiveness, chronic application of WAS for 10 days provoked colonic hyperalgesia, increased colonic motility and caused local inflammation. Moreover, the colonic signs persisted for the following 20 days [37]. The main limit of this model regards its susceptibility to several factors such as animal strain, sex and environmental conditions that could affect the results [8,16]. To synthesize, the WAS model carries both face- and construct-validity [23].

Lesser known and still a matter of investigation is the possibility that the two models of chronic stress presently reviewed possess also the predictive validity i.e., how well the models are responsive to treatment showing some efficacy in alleviating visceral pain to predict triggered treatment. Noteworthy, WAS shows some peculiarities, i.e., the absence of a tolerance after repeated applications and the maintenance of hyperalgesia and hypermotility after the stress is removed, that make it particularly suitable to investigate the ability of drugs to prevent or attenuate the appearance and evolution of the colonic alterations induced by stress, and also to evaluate long lasting beneficial effects of the drug after its withdrawal.

## 3. The Enteric Neurotransmission as a Potential Target of IBS Treatment

It has been suggested that pain signals originate in intrinsic primary afferent neurons and are transmitted by extrinsic primary afferent neurons [38]. Afferent nerve endings are endowed with a variety of pro- and antinociceptive ion channels and receptors; the balance between pain sensing and suppressing signals finally determines their activation status [38]. Important neurotransmitters involved in visceral sensation are CRF, 5-HT and neurokinins; channels mediating activation of afferent nerves are e.g., transient receptor potential (TRP) ion channels, that act as molecular detectors of thermal and chemical stimuli that activate sensory neurons to produce acute or persistent pain. Due to the recent development of drugs approved for IBS, guanylate cyclase (GC) receptors and chloride channels have received much attention.

### 3.1. Cortocotropin Releasing Factor (CRF)

**Figure 2:**
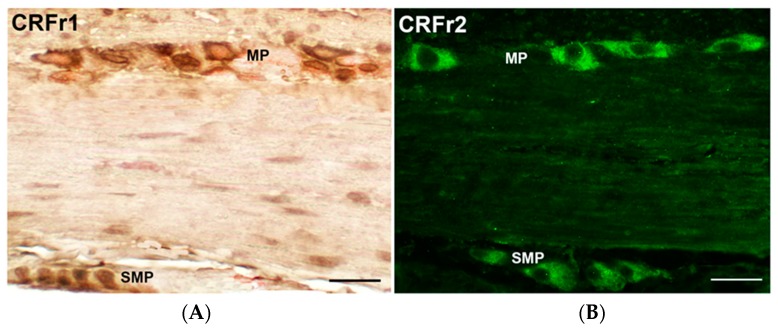
(**A**): CRFr1 and (**B**): CRFr2-immunoreactivity in the myenteric and submucous plexuses of rat colon. Bar = 40 μm.

Psychological stress at any point of the lifetime can bring permanent alterations of several systems such as the descending pain modulatory system, the immune system, and the gut microbiota. Preclinical evidence has accumulated over the years suggesting that stress-related alterations of colonic motor and visceral functions are primarily mediated by the activation of the brain CRFr/CRF1 signaling pathway, while CRF2 receptor activation may exert a counteracting influence [39,40,41]. CRFr are located in effector neurons of the hypothalamus, the amygdala, the cingulate cortex and the locus caeruleus complex. Central injection of CRF can reproduce behavioral and physiological reactions similar to those seen in response to acute psychological stress [42,43], and inhibition of CRF-mediated responses by antagonists [43,44] or in knockout animals results in a decrease of the animals’ answer to stress [45,46]. However, recent experimental studies point to an equally important contribution of the gut CRFr1-2/CRF signaling to the gastrointestinal (GI) stress response [21,47]. In the GI tract the CRFr are expressed by enteric neurons (Figure 2) and mucosal cells [20,21,48,49,50,51,52] and an upregulation of these receptors has been reported in the colon of male Wistar rats underwent to delayed stress-induced visceral hyperalgesia [37,44] and WRS [20,21].

Although CRF has been the subject of 33 years of effort in research by academia and the pharmaceutical industry that resulted in several thousand papers and patents, yet little progress has been made to identify and market diagnostic or therapeutic CRF peptides and small molecule ligands, and no modulators of this peptide has reached the pharmaceutical market for IBS so far [53].

### 3.2. The Serotonin or 5-Hydroxytriptamine (5-HT)

Several lines of evidence indicate that altered 5-HT signaling in the central nervous system and in the gut contributes to the pathophysiology in IBS [54]. Further, patients with IBS-diarrhea (IBS-D) have increased postprandial plasma 5-HT, while those with IBS-constipation (IBS-C) have reduced postprandial 5-HT levels [55]. 5-HT released from enterochromaffin cells regulates sensory, motor and secretory functions of the digestive system through the interaction with different receptor subtypes. A recent experimental work [56] has shown that 5-HT3r antagonist alosetron and the CRF1r antagonist E2508 reduced defecation and visceral pain in WRS-subjected rats. Nevertheless, the anti-serotoninergic drugs that have been introduced on the market such as tegaserod, a 5-HT-4 agonist, and the same alosetron, seem to be effective only in a limited part of the IBS patients and suffer from the evidence of side effects (ischemic colitis, diarrhea, cardiovascular ones) that has let the Food and Drug Administration to impose a limitation of their widest use (https://www.alosetronrems.com/AlosetronUI/rems/pdf/letterforhealthcareproviders.pdf; https://www.fda.gov/Drugs/DrugSafety/ucm103223.htm). Newer 5-HT4 agonists such as prucalopride, naronapride, velusetrag, and YKP10811 have been found active only in chronic idiopathic constipation. Further studies are needed in order to develop an optimal treatment in the field of serotonin.

### 3.3. Tachykinins

NK2r antagonists have been proven effective in animal models of visceral hyperalgesia induced by inflammation [57,58] or WAS [59]. Nepadutant [58], ibodutant [57] or saredutant [59] were found able to inhibit rectal hypersensitive responses in rats, guinea-pigs or gerbils pretreated with Trinitrobenzenesulfonic acid (TNBS) or previously subjected to restraint, suggesting that the NK2r has a role in mediating visceral allodynia/hyperalgesia. This role was further supported by the studies of Birder et al. [60], who showed that the increased expression of either c-fos and c-jun proto-oncogene markers in spinal cord and dorsal root ganglia neurons of rats pretreated with TNBS was prevented by nepadutant, and by Laird et al. [61], who found nepadutant capable of preventing the hypersensitivity of single spinal cord neurons responding to colorectal distension or pelvic nerve stimulation in rats pretreated with intracolonic acetic acid. Laird et al. [62] have also shown that intracolonic instillation of acetic acid or capsaicin fails to produce either acute (cardiovascular) responses or primary hyperalgesia in NK1r-knockout mice, thus suggesting a role for even this latter receptor, as mediator of visceral hyperalgesia. The NK1r antagonist aprepitant effectively reduced defecation and CD as well as increased adrenocorticotropin in gerbils subjected to WRS [59]. Conversely, a recent study suggests that the SP system might play little role in the development of visceral hyperalgesia in the neonatal maternal separation (MS) rat model [63]. We found a decrease in myenteric NK1-immunoreactive (IR) neurons and a decrease in Substance P-IR nerve fibers in the muscle wall after WRS [19].

Also, the NK3r (located at peripheral or/and central level) could play a role in visceral hyperalgesia, due to the reported effectiveness of either intraperitoneal [64] or intrathecal [65] administration of the NK3r selective antagonist SR 142801 in reducing reflex abdominal contractions elicited by various nociceptive stimuli. Gender and species differences are reported in the modulating effect on pain of tachykinin antagonists [57,66,67].

In spite of these promising experimental results, none of these NKr antagonists have reached the market due to limitation of their effect in humans and the presence of side effects found in the development clinical phases.

### 3.4. Calcium Channels

It has been reported that T-type calcium channels encoded by the Ca(V)3.2 isoform are expressed in colonic nociceptive primary afferent neurons and that they contribute to the exaggerated pain perception in a butyrate-mediated rodent model of IBS [68]. These preliminary results render T-type antagonists as potential candidates for the development of novel therapeutic agents in the treatment of IBS. A modulation of T-type calcium channels has been reported for the antispasmodic otilonium bromide (OB) [69]. OB is a quaternary ammonium derivative poorly absorbed from the GI tract, that concentrates mainly in the large intestine acting locally as an L-type calcium channel blocker, an antimuscarinic and a NK2r antagonist [70]. Probably thanks to this multi-target activity, OB effectively reduced pain and improved defecation alterations in placebo-controlled trials in IBS patients [71]. Conversely, OB tested in experimental models of visceral sensitivity, at least in WRS, was less active as compared to the other agents previously reviewed [21]. Other antispasmodics such as pinaverium bromide endowed with L-type calcium channel blocker and antimuscarinic activity improved motility disorders and consequently reduced stool problems in IBS patients.

### 3.5. Others Receptors and Channels

Since irritation, immune challenge and inflammation releases both tachykinin and calcitonin gene-related peptide (CGRP) from extrinsic afferents and from intrinsic neurons inside the gut, these peptides may either increase the peripheral sensory gain of extrinsic afferents or contribute to the afferent transmission of algetic signals within the central nervous system. As gut extrinsic afferents do not possess receptors for CGRP, it is probable that peptide-induced sensitization or excitation of sensory neurons is an indirect consequence of peptide action on gastrointestinal effectors [38]. In experimental models of colorectal distension-induced visceral pain, CGRP has been shown to play a role [72]. In rats underwent to MS, it has been found that the high number of mast cells associated with an increase in CGRP-IR [28]; similarly, an increase in CGRP expression and a higher number of mast cells were described in the mucosa and submucosa of rats underwent to WRS [20]. Noteworthy, in rectal biopsy from patients with well characterized rectal hypersensitivity, a marked increase in CGRP was found [73].

The increase in the vanilloid receptor TRPV1 significantly correlated with the decrease in heat and distension sensory threshold confirming that both afferent and intrinsic fibers take part in the change of signaling pathways of visceral hypersensitivity. Further, WAS could induce the upregulation of TRPV1 and TRPA1 in the colonic afferent DRG indicating these receptors as candidate molecules in stress-induced hyperalgesia in rats [74]. An antispasmodic largely used in the United States for IBS such as peppermint oil, whose active component is menthol, has recently been found antinociceptive thanks to its capability to activate temperature sensing ion channel TRPM8 [75].

**Figure 3:**
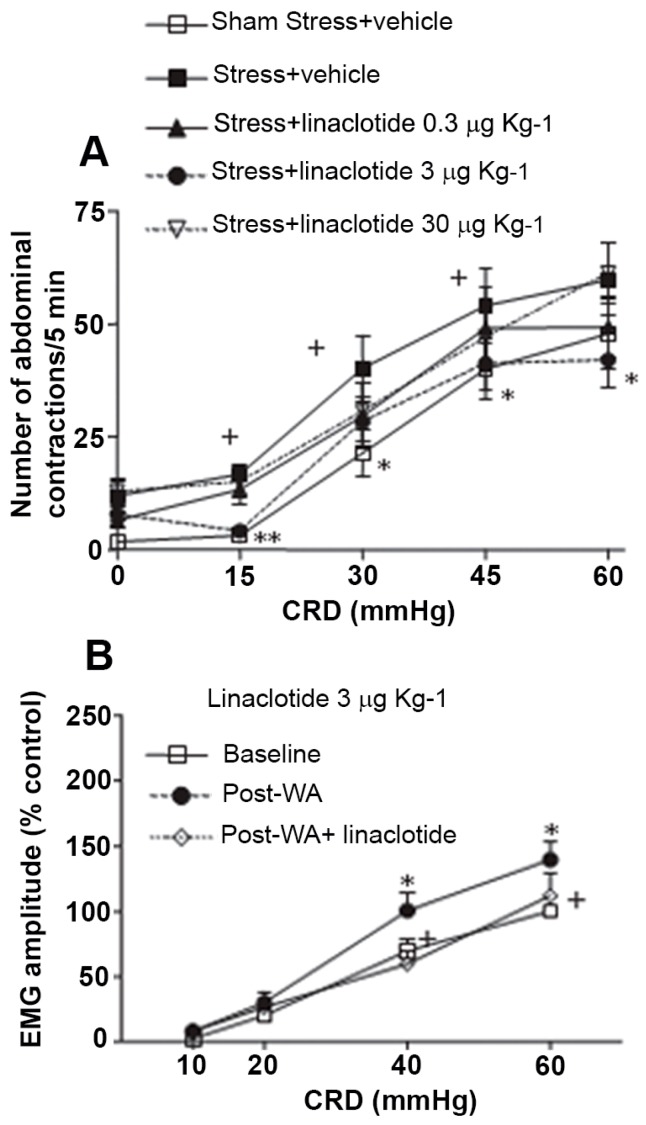
(**A**): Effect of oral administration of linaclotide (0.3, 3 or 30 microg/kg) or vehicle on acute restraint stress-induced colorectal hypersensitivity to colorectal distension (CRD). (**B**): Effect of acute water avoidance stress on electromyografic response (EMG) to CRD. An acute session of water avoidance stress (WAS)-induced increased EMG response 24 h later. Linaclotide at 3 microg/kg p.o. completely abolished stress-induced hyperalgesia. Modified from Eutamene et al. [76]. (**A**) + = *p* < 0.05 vs. sham-stress + vehicle animals. * = *p* < 0.05 vs. stressed animals treated with vehicle and (**B**) * = *p* < 0.05 significantly different from baseline, + = *p* < 0.05 significantly different from pre-WAS.

It should be noted that while modulators of vanilloid receptors are under preclinical development, activators of guanylate cyclase (GC) receptor has produced a drug successfully introduced in the market for IBS with predominant constipation (IBS-C) such as linactotide. Linaclotide has shown potent anti-nociceptive effects in several mechanistically different rodent models of visceral hypersensitivity (WRS, WAS, inflammatory conditions; see Figure 3) both in Wistar rats and GC-null mice [76]. It activates the GC-C expressed on mucosal epithelial cells, resulting in the production and release of cGMP. This extracellular cGMP acts on and inhibits nociceptors, thereby reducing nociception [77]. In this area another GC activator such as plecanatide will be reviewed by the FDA for approval in IBS-C indication in the first quarter of 2018 [17].

Another drug approved for IBS-C is lubiprostone, a chloride channel activator which acts locally on the apical membrane of epithelial cells in the gastrointestinal lumen. As a derivative of prostaglandin E1, lubiprostone selectively combines with prostaglandin receptor to activate chloride channel 2 leading to chloride-rich fluid secretion. Increasing fluid secretion softens stools and improves intestinal motility, which finally contributes to alleviate constipation symptoms of IBS-C [17].

Tenapanor is a small-molecule inhibitor of the gastrointestinal sodium/hydrogen exchanger NHE3, a protein present in the apical side of enterocytes, which results in increased intraluminal sodium and water excretion and is in active development for IBS-C [78].

Translocator protein 18 kDa (TSPO) is a five-domain transmembrane protein that is highly expressed in steroid-producing tissues, including the glial cells within the brain. ONO-2952 is a novel and selective inhibitor of TSPO that reduces stress-induced defecation and visceral hyperalgesia in rat models [79].

Other classes of drugs challenged in IBS are those acting on opioid or GABA receptors. Among opioid agonists, loperamide and diphenoxylate are anti-diarrheal agents that have been used in IBS for many years but their efficacy is already discussed and not completely proven while the recent one eluxadoline has been licensed in USA for IBS-D with some limitations due to its side effects (https://www.fda.gov/Drugs/DrugSafety/DrugSafetyPodcasts/ucm547907.htm), GABAergic agents are alpha2β ligands that generally bind potently to an auxiliary protein associated with voltage-gated calcium channels, reducing depolarization-induced calcium influx at nerve terminals. This reduces the release of several excitatory neurotransmitters, including glutamate, noradrenaline, substance P, and calcitonin gene-related peptide (CGRP), which are involved in pain mechanisms. Gabapentin and pregabalin did not show a good efficacy/risk ratio in clinical trials.

## 4. Conclusions

Notwithstanding the limits of the IBS experimental models as compared to the complex picture characterizing the human manifestations, these models have allowed to confirm the consistent role of psychosocial stressors in the genesis and time-course of the gut symptomatology. The models reviewed (WRS, WAS, MS) are able to induce visceral hypersensitivity that is one of the main signs of IBS. To translate the visceral hypersensitivity measured in the animals to the abdominal human pain is not so likely. Several differences in pain threshold, central processing of nociceptive modulation, emotional and cognitive contributions between rodents and humans limit this translation. However, the three animal models have demonstrated that numerous neurotransmitters are involved in this nociception and that modulators of them are generally active in reducing experimental pain. Nevertheless, because of the complexity of this phenomenon in humans, only few of these latter molecules have been found active in clinical studies and this has hampered the development of new drugs for IBS. 

Up to date, the focus on targets related to specific symptoms has brought the production of drugs aimed to treat constipation or diarrhea with alternate results. This choice did not have attention on the possibility to look for drugs useful for global IBS symptoms. A more integrated view on the disease seems to be desirable for now, in order to develop efficient therapies that can counteract the chronic natural history of IBS.
